# Supplementary material for: Perception of People Living with HIV and Healthcare Workers on Differentiated Service Delivery Programs in Nigeria: A Qualitative Study
Source: PLoS One. 2025 May 27;20(5):e0309254. doi: 10.1371/journal.pone.0309254 (PMC12111639; doi:10.1371/journal.pone.0309254)
Supplement: Attachment — Submitted filename: PONE-D-24-32833_reviewer.pdf [file pone.0309254.s001.pdf]

PLOS ONE

# Perception of People Living with HIV and Healthcare Workers on Differentiated Service Delivery Programs in Nigeria: A Qualitative Study

--Manuscript Draft--

| | |
|---|---|
| **Manuscript Number:** | PONE-D-24-32833 |
| **Article Type:** | Research Article |
| **Full Title:** | Perception of People Living with HIV and Healthcare Workers on Differentiated Service Delivery Programs in Nigeria: A Qualitative Study |
| **Short Title:** | Perception of PLHIV and Healthcare Workers on Differentiated Service Delivery Programs in Nigeria |
| **Corresponding Author:** | Adewale Akinjeji, MD<br>Karolinska Institute: Karolinska Institutet<br>SWEDEN |
| **Keywords:** | |
| **Abstract:** | Nigeria, home to approximately 1.9 million people living with HIV (PLHIV), faces significant challenges in providing adequate care and treatment to the teeming number of PLHIV, particularly following the adoption of the ‘test and treat’ policy and the HIV ‘SURGE’ initiative. The Differentiated Service Delivery (DSD) model was introduced to address the increased burden on the healthcare system, offering patient-centered care through diverse methods like community-based care, fast-track drug refills, and task-shifting to lower-level healthcare providers. This study, conducted by the National AIDS and STI Control Program (NASCP) in collaboration with ICAP at Columbia University, evaluates the perspectives of healthcare workers and PLHIV on DSD programs in Nigeria.<br>This is a qualitative study involving key informant interviews (KIIs) with 12 healthcare workers and focus group discussions (FGDs) with 153 PLHIV across four Nigerian states (Anambra, Kaduna, Lagos, Taraba). Healthcare workers with over one year of ART service experience and PLHIV on ART for at least 12 months with suppressed viral loads were included in the study. Data was analyzed using an inductive, thematic approach to identify emergent patterns and themes.<br>The study revealed that DSD models, such as peer-led Facility ART groups and community pharmacy ART refills, were widely implemented. The primary factors influencing the deployment of DSD models included client convenience and the need to alleviate healthcare worker load. Positive experiences with DSD were associated with convenience, confidentiality, and affordability, whereas negative perceptions stemmed from high service costs, poor healthcare worker attitudes, and confidentiality concerns. Additionally, the study highlighted the necessity for continuous training of healthcare workers, engagement with PLHIV, and increased awareness programs to improve DSD service delivery.<br>Overall, the study underscores the importance of addressing financial barriers, enhancing healthcare worker training, and ensuring confidentiality in optimizing DSD models and improving HIV care outcomes in Nigeria. |
| **Order of Authors:** | Johnbaptist Ezenduka, MD |
| | Peter Nwaokennaya |
| | Gbenga Benjamin Obasa |
| | Geoffrey Ogbeke |
| | Ogonna Onuorah |
| | Lawal Abubakar |
| | Adebobola Bashorun |
| | Ginigeme Ogochukwu |
| | Emerenini Franklin |
| | Adewale Akinjeji |

Powered by Editorial Manager® and ProduXion Manager® from Aries Systems Corporation

| Additional Information: | |
|---|---|
| **Question** | **Response** |
| **Financial Disclosure**<br><br>Enter a financial disclosure statement that describes the sources of funding for the work included in this submission. Review the submission guidelines for detailed requirements. View published research articles from *PLOS ONE* for specific examples.<br><br>This statement is required for submission and **will appear in the published article** if the submission is accepted. Please make sure it is accurate.<br><br>**Funded studies**<br>Enter a statement with the following details:<br>• Initials of the authors who received each award<br>• Grant numbers awarded to each author<br>• The full name of each funder<br>• URL of each funder website<br>• Did the sponsors or funders play any role in the study design, data collection and analysis, decision to publish, or preparation of the manuscript?<br><br>Did you receive funding for this work? | The author(s) received no specific funding for this work. |
| **Competing Interests**<br><br>Use the instructions below to enter a competing interest statement for this submission. On behalf of all authors, disclose any competing interests that could be perceived to bias this work—acknowledging all financial support and any other relevant financial or non-financial competing interests.<br><br>This statement is required for submission and **will appear in the published article** if the submission is accepted. Please make sure it is accurate and that any funding sources listed in your Funding Information | The authors have declared that no competing interests exist. |

| | |
|---|---|
| later in the submission form are also declared in your Financial Disclosure statement.<br><br>View published research articles from *PLOS ONE* for specific examples.<br><br>**NO authors have competing interests**<br><br>Enter: *The authors have declared that no competing interests exist.*<br><br>**Authors with competing interests**<br><br>Enter competing interest details beginning with this statement:<br><br>*I have read the journal's policy and the authors of this manuscript have the following competing interests: [insert competing interests here]*<br><br>* typeset | |
| **Ethics Statement**<br><br>Enter an ethics statement for this submission. This statement is required if the study involved:<br><br>• Human participants<br>• Human specimens or tissue<br>• Vertebrate animals or cephalopods<br>• Vertebrate embryos or tissues<br>• Field research<br><br>Write "N/A" if the submission does not require an ethics statement.<br><br>General guidance is provided below. Consult the submission guidelines for detailed instructions. **Make sure that all information entered here is included in the Methods section of the manuscript.** | National Health Research Ethics Committee of Nigeria (NHREC)<br><br>NHREC Protocol Number NHREC/01/01/2007- 19/07/2023<br>NHREC Approval Number NHREC/01/01/2007- 27/07/2023<br>Date: 27<br>th July, 2023<br><br>Written consent was obtained from all the participants |

| | |
|---|---|
| **Format for specific study types**<br><br>**Human Subject Research (involving human participants and/or tissue)**<br>• Give the name of the institutional review board or ethics committee that approved the study<br>• Include the approval number and/or a statement indicating approval of this research<br>• Indicate the form of consent obtained (written/oral) or the reason that consent was not obtained (e.g. the data were analyzed anonymously)<br><br>**Animal Research (involving vertebrate animals, embryos or tissues)**<br>• Provide the name of the Institutional Animal Care and Use Committee (IACUC) or other relevant ethics board that reviewed the study protocol, and indicate whether they approved this research or granted a formal waiver of ethical approval<br>• Include an approval number if one was obtained<br>• If the study involved *non-human primates*, add *additional details* about animal welfare and steps taken to ameliorate suffering<br>• If anesthesia, euthanasia, or any kind of animal sacrifice is part of the study, include briefly which substances and/or methods were applied<br><br>**Field Research**<br><br>Include the following details if this study involves the collection of plant, animal, or other materials from a natural setting:<br>• Field permit number<br>• Name of the institution or relevant body that granted permission | |
| **Data Availability**<br><br>Authors are required to make all data underlying the findings described fully available, without restriction, and from the time of publication. PLOS allows rare exceptions to address legal and ethical concerns. See the PLOS Data Policy and FAQ for detailed information. | Yes - all data are fully available without restriction |

Powered by Editorial Manager® and ProduXion Manager® from Aries Systems Corporation

| | |
|---|---|
| A Data Availability Statement describing where the data can be found is required at submission. Your answers to this question constitute the Data Availability Statement and **will be published in the article**, if accepted.<br><br>**Important:** Stating 'data available on request from the author' is not sufficient. If your data are only available upon request, select 'No' for the first question and explain your exceptional situation in the text box.<br><br>Do the authors confirm that all data underlying the findings described in their manuscript are fully available without restriction? | |
| **Describe where the data may be found in full sentences. If you are copying our sample text, replace any instances of XXX with the appropriate details.**<br><br>• If the data are **held or will be held in a public repository**, include URLs, accession numbers or DOIs. If this information will only be available after acceptance, indicate this by ticking the box below. For example: *All XXX files are available from the XXX database (accession number(s) XXX, XXX.).*<br>• If the data are all contained **within the manuscript and/or Supporting Information files**, enter the following: *All relevant data are within the manuscript and its Supporting Information files.*<br>• If neither of these applies but you are able to provide **details of access elsewhere**, with or without limitations, please do so. For example:<br><br>*Data cannot be shared publicly because of [XXX]. Data are available from the XXX Institutional Data Access / Ethics Committee (contact via XXX) for researchers who meet the criteria for access to confidential data.*<br><br>*The data underlying the results presented in the study are available from (include the name of the third party* | All relevant data are within the manuscript and its Supporting Information files. |

| | |
|---|---|
| *and contact information or URL).*<br>• This text is appropriate if the data are owned by a third party and authors do not have permission to share the data.<br><br>* typeset | |
| Additional data availability information: | |

Manuscript
Click here to access/download;Manuscript;DSD_ perception study_updated_final.docx

# Perception of People Living with HIV and Healthcare Workers on Differentiated Service Delivery Programs in Nigeria: A Qualitative Study

Authors: Johnbaptist Ezenduka[a], Peter Nwaokennaya[b], Gbenga Benjamin Obasa[a], Geoffrey Ogbeke[b], Ogonna Onuorah[a], Lawal Abubakar[a], Adebobola Bashorun[b], Ginigeme Ogochukwu[a], Emerenini Franklin[a], Adewale Akinjeji[a,c].
a: ICAP Nigeria, Abuja, Nigeria
b: National AIDS and STI Control Program, Ministry of Health, Abuja, Nigeria
c. Department of Global Public Health, Karolinska Institutet, Stockholm, Sweden
Corresponding author: Adewale Akinjeji adewale.akinjeji@ki.se

## Abstract

Nigeria, home to approximately 1.9 million people living with HIV (PLHIV), faces significant challenges in providing adequate care and treatment to the teeming number of PLHIV, particularly following the adoption of the 'test and treat' policy and the HIV 'SURGE' initiative. The Differentiated Service Delivery (DSD) model was introduced to address the increased burden on the healthcare system, offering patient-centered care through diverse methods like community-based care, fast-track drug refills, and task-shifting to lower-level healthcare providers. This study, conducted by the National AIDS and STI Control Program (NASCP) in collaboration with ICAP at Columbia University, evaluates the perspectives of healthcare workers and PLHIV on DSD programs in Nigeria.

This is a qualitative study involving key informant interviews (KIIs) with 12 healthcare workers and focus group discussions (FGDs) with 153 PLHIV across four Nigerian states (Anambra, Kaduna, Lagos, Taraba). Healthcare workers with over one year of ART service experience and PLHIV on ART for at least 12 months with suppressed viral loads were included in the study. Data was analyzed using an inductive, thematic approach to identify emergent patterns and themes.

The study revealed that DSD models, such as peer-led Facility ART groups and community pharmacy ART refills, were widely implemented. The primary factors influencing the deployment of DSD models included client convenience and the need to alleviate healthcare worker load. Positive experiences with DSD were associated with convenience, confidentiality, and affordability, whereas negative perceptions stemmed from high service costs, poor healthcare worker attitudes, and confidentiality concerns. Additionally, the study highlighted the necessity for continuous training of healthcare workers, engagement with PLHIV, and increased awareness programs to improve DSD service delivery.

Overall, the study underscores the importance of addressing financial barriers, enhancing healthcare worker training, and ensuring confidentiality in optimizing DSD models and improving HIV care outcomes in Nigeria.

## Introduction

Nigeria is among the countries that have the highest burden of HIV globally. With an estimated 1.9 million people living with HIV (PLHIV), effective management and treatment remain critical priorities for public health initiatives in the country[1]. In 2016, Nigeria adopted the 'test and treat' which stipulates a rapid ART initiation for all persons who test HIV positive, and the subsequent implementation of the HIV 'SURGE' resulted in a rapid scale-up of HIV case finding and increased number of Persons Living with HIV placed on treatment thereby placing a higher-than-usual burden on the fragile healthcare system.[2,3,4,5] Differentiated Service Delivery (DSD) model of care was among the innovative approaches adopted to address the challenges associated with a high volume of persons in HIV care and treatment in a country with scarce resources, [6].

Differentiated Service Delivery programs is a Patient-centered approach to HIV care, aiming to tailor services to the diverse needs of PLHIV.[7] These include various models, such as community-based care, fast-track drug refills, and task-shifting responsibilities to lower-level healthcare providers.[8], DSD programs seek to enhance treatment adherence, Continuity of treatment, and overall health outcomes for PLHIV while alleviating the strain on healthcare systems by offering flexible service delivery options. [9,10] Nigeria officially adopted the DSD approach in line with WHO recommendations in 2019 and has since scaled the implementation across the 36+1 subnational

units of the federation.[11] The rapid scale-up of DSD contributed to the resilience of the HIV program in-country during the COVID-19 pandemic.[11] The COVID-19 pandemic and the resulting global response reinforced the need for countries to adopt and evolve sustainable mechanisms for managing chronic conditions, including ART for PLHIV.

Despite the potential benefits of DSD programs[12], there is a need to regularly review the effectiveness of services and activities in these settings. A critical aspect of evaluating the effectiveness of DSD involves assessing the perceptions and experiences of both PLHIV and healthcare professionals (HCWs)PLHIV. Understanding their perspectives is crucial for identifying barriers to access, adherence, and successful program implementation[13]. Therefore, exploring the perceptions of PLHIV and HCWs regarding DSD programs in Nigeria is essential for informing policy decisions, improving program design, and ultimately advancing the quality of HIV care and treatment services in the country.

The National AIDS and STI Control Program (NASCP) of the Nigeria Federal Ministry of Health collaborated with ICAP at Columbia University to assess the perspectives of healthcare professionals and PLHIV on Differentiated Service Delivery programs in Nigeria to gain comprehensive insights into the efficacy and impact of these programs on HIV care and treatment in the country.

## Methodology

### Setting and Recruitment

This qualitative study, part of a larger mixed-methods investigation, aimed to understand contextual factors influencing differentiated service delivery (DSD) models' uptake and retention in four socio-culturally diverse Nigerian states (Anambra, Kaduna, Lagos, Taraba). Participant experiences were captured through key informant interviews with healthcare workers and focus group discussions with people living with HIV.

Qualitative data were collected through semi-structured interviews and discussions, employing audio recording, transcription, and confidentiality protocols. Interview recruitment commenced on 14 August 2023 and ended on 25 August 2023.

#### Key Informant Interviews

Twelve key informant interviews were conducted with healthcare workers providing HIV services in Anambra, Kaduna, Lagos, and Taraba states (3 per state). Healthcare providers with more than One year of experience providing ART services were purposively selected as Key informants. Trained interviewers used a semi-structured guide to facilitate in-depth discussions on differentiated service delivery implementation and service provision. The interviews aimed to elicit comprehensive insights from frontline workers operationalizing DSD models, allowing exploration of predefined and emerging topics while ensuring consistency through the interview guide and interviewer training.

#### Focus Group Discussions

Sixteen FGDs involving 153 participants were conducted, stratified into key populations (4), general adult population stratified by gender (8), and young adults aged 18-24 years (4). Participants had been on ART for ≥12 months with suppressed viral loads. FGDs explored experiences, challenges, and recommendations related to DSD models. Recruitment for KIIs and FGDs followed standardized procedures, with eligibility screening and assurances of voluntary participation without adverse impacts.

### Inclusion and exclusion criteria

#### Inclusion Criteria

#### Key Informant Interviews

Key informants who are adults (Aged 18 years) have provided HIV services for at least one year and consent to participate.

#### Focus Group Discussions

PLHIV over the age of 18 years who received care in the selected facilities and expressed their willingness to participate were considered eligible for inclusion. Also, participants must have been on ART for at least 12 months before the interview date and had achieved a suppressed viral load at their last assessment.

**Exclusion Criteria**

Healthcare workers and PLHIV who did not consent to participate in the study were excluded from the qualitative analysis. To ensure the data's integrity and reliability, individuals who failed to respond to survey questions during the data collection process were also excluded from the study.

**Data Analysis**

The analysis followed an inductive, thematic approach, identifying emergent patterns and themes. Peer analysis, consensus discussions, and iterative coding of responses ensured rigor and reliability. The quantitative analysis involved descriptive statistics and thematic coding using Dedoose software, with a priori and emergent codes based on study objectives. Data were organized hierarchically to uncover related explanations and patterns.

**Ethical Consideration and Informed Consent**

Before data collection commenced, ethical approval was obtained from the Institutional review boards in Nigeria (NHREC/01/01/2007). Informed consent was obtained from all participants, ensuring confidentiality and voluntary participation. Participants were informed about the study's purpose and rights, and only those who provided consent were included.

## RESULTS/FINDINGS

**Key Informant Interview (KII) Analysis**

A total of 12 respondents participated in the KII, with an equal distribution of gender, comprising 50% male and 50% female. Among them, 33% were physicians, 25% were nurses, 17% were case managers and pharmacists, and 8% were public health specialists. Regarding experience providing ART services, 42% have less than five years, 33% between 6 to 10 years, and 25% more than ten years.

### Table 1. Demographics Characteristics

| Responses | N**(%)** |
|---|---|
| **Category** | |
| Male | 6(50%) |
| Female | 6(50%) |
| Physicians | 4(33%) |
| Nurses | 3(25%) |
| Case Managers | 2(17%) |
| Pharmacists | 2(17%) |
| Public Health Specialists | 1(8%) |
| Less than 5 years | 5(42%) |
| 6 to 10 years | 4(33%) |
| More than 10 years | 3(25%) |

**DSD Coverage**

The availability of DSD models in facilities varied. The most common models across most facilities were the peer-led Facility ART group, fast track, and mother-infant pair/mentor mother-infant pair. They were followed by.

Community pharmacy ART refill. The other models varied in their availability across sites, as shown in Table 2 below.

**Table 2. DSD Coverage**

| DSD Coverage | N(%) |
|---|---|
| Facility ART group support (ground led) | 11(92%) |
| Fast track | 10(83%) |
| Mother-infant pair/mentor mother-infant pair | 9(75%) |
| Community pharmacy ART refill | 8(67%) |
| Facility child/teen/adolescent club | 7(58%) |
| Home delivery | 7(58%) |
| Weekend and public holidays | 7(58%) |
| Community ART refill group HCW led | 6(50%) |
| Community ART refill group PLHIV led | 6(50%) |
| Facility ART group HCW led | 5(42%) |
| After Hours | 5(42%) |
| One-Stop Shop (OSS) | 5(42%) |
| Adolescent community ART/Peer-led groups | 4(33%) |
| Decentralized (hub and spoke) | 2(17%) |

**Deployment of DSD Models**

Several factors influenced the decision of healthcare workers and facilities to deploy different DSD models, and these are presented in Table 3. The results show that client convenience and the need to reduce the healthcare worker load significantly influenced the model deployed in the facilities. However, the client's eligibility for a specific model and the need to improve patient care were of the slightest significance. Other Factors considered included distance to facilities, cost of transportation, and the need to address stigma.

**Table 3. DSD Deployment**

| Deployment of DSD Models | N(%) |
|---|---|
| Client convenience | 6(50%) |
| Alleviating facility workload | 6(50%) |
| Long proximity to facilities | 3(25%) |
| Best fit for the clients | 2(17%) |
| Clients Eligibility | 1(8%) |
| Client Schedule | 2(17%) |
| Tailoring care to client needs and clinical staging | 2(17%) |
| Addressing transportation costs | 2(17%) |
| Transportation | 2(17%) |
| Minimizing stigmatization | 2(17%) |
| Improve Patients Care | 1(8%) |

**Decision to Deploy**

Reasons for devolution of HIV/AIDS care services include prioritizing client convenience (50%), alleviating facility workload (50%), reducing long proximity to facilities (25%), tailoring care to fit client needs and clinical staging (16.67%), addressing transportation costs (16.67%), and minimizing stigmatization (16.67%).

**Reasons for declining Devolvement**

Healthcare workers provided some reasons clients have either declined devolvement or voluntarily opted to be returned to a routine standard of care following devolvement to a DSD model. The most common reasons for this included the cost of services at the DSD service delivery points and confidentiality concerns. Other factors include poor HCW attitude, a preference for facility services, proximity, and stigmatization.

**Focus Group Discussions**

The Focus Group Discussions (FGDs) convened a total of 153 participants, consisting of 79 males (51.65%) and 74 females (48.35%), providing a balanced gender representation. Respondent characteristics presented in Table 6 show that most respondents are adults (above 18 years) who have been on ART for a minimum of 1 year and are familiar with DSD models. This is in line with the recommendations in the Nigeria guidelines for DSD eligibility (Ref guideline)

**Table 4: FGD Demographics**

| Responses | N= 153(%) |
|---|---|
| **Category** | |
| Males | 79(51.65%) |
| Females | 74(48.35%) |
| Lagos | 40(26.14%) |
| Taraba | 39(25.49%) |
| Anambra | 38(24.84%) |
| Kaduna | 36(23.53%) |
| Men | 40(26.14%) |
| Key Population | 39(25.49%) |
| Women | 38(24.84%) |
| Adolescents | 36(23.53%) |
| 18-24 years | 33(21.57%) |
| 25-39 years | 39(25.49%) |
| 40-49 years | 45(29.41%) |
| 50-64 years | 31(20.26%) |
| 65 and Above | 2(1.31%) |
| Decline | 3(1.96%) |
| Completed Secondary School | 63(41.18%) |
| Completed College of Education/Polytechnic | 45(29.41%) |
| Completed University | 28(18.00%) |
| Completed Post- Graduate Degree | 7(5.00%) |
| Completed Primary School | 6(4.00%) |
| Declined | 3(2.00%) |
| Never been to School | 1(0.41%) |

| | |
|---|---|
| Between 1 and 5 Years | 38(25%) |
| Between 6 and 10 Years | 44(30%) |
| Above 10 years | 65(42.%) |
| Declined to answer | 5(3.%) |
| Yes | 120(79.00%) |
| No | 32(21.00%) |

**Themes, Codes, and Exemplar Quotes**

The responses from the PLHIV were categorized into two main themes: barriers and enablers to the uptake of DSD services. These responses were then further divided into specific sub-themes presented below.

**Theme 1: <u>Enablers</u>**

The participants discussed factors contributing to a positive experience at DSD service delivery points, including convenience, confidentiality, and reduced service access costs. These factors promoted their uptake of DSD models and varied in their level of importance to the participants.

**Convenience**

This was most important in shaping positive experiences for men and young adult participants. Factors contributing to increased convenience included proximity to pick-up points, flexible appointment scheduling, and streamlined processes. These factors significantly improved the participants' access to and utilization of HIV services. As one of the Men puts it, *"I am currently enjoying the DSD program because they have been delivering my medications to my home for a while now. Due to my busy schedule, I often cannot make it to the facility in person, but I still go for my viral load tests."*

**Confidentiality**

Confidentiality emerged as a key theme for the key population and women participants. This underscores the critical role of privacy in healthcare settings, which can foster trust and encourage individuals to seek and adhere to HIV-related care and treatment schedules. The confidential setting in some DSD service delivery points encouraged the uptake of services and retention in care. A member of the key population said, *"Home delivery is a better option because it prevents you from having to visit a community center to collect your medication. This way, you can avoid the possibility of running into someone you know. So, to avoid any potential embarrassment, home deliveries are a better option."*

**Affordability**

Service affordability also emerged as another key theme that shaped the positive perception of care, especially among women. Affordability, in this case, refers to the cost of paying for the services and the cost of transport to the service delivery point. This suggests that removing financial barriers to healthcare access can significantly improve individuals' ability to seek and engage in HIV care. Initiatives to alleviate these costs are therefore highly valued by service users. A female respondent said, *"I receive home delivery, which helps reduce my transportation and the associated stress."*

**Theme 2: <u>Barriers</u>**

Exploring the factors that can negatively influence the perception of DSD services among PLHIV showed varied responses from the participants. The key factors influencing the negative perception of PLHIV ranged from the high cost of services to confidentiality issues and the poor attitude of healthcare workers. These unpleasant experiences were experienced at the facility and community DSD models and negatively influenced participants' willingness to accept DSD and be retained in DSD service points.

**Cost of services**

The cost of services was significantly expressed as contributing to the negative perception experienced by adult male and female respondents. They highlighted the financial burden associated with accessing HIV services, including testing, treatment, and medications in some DSD service delivery points and health facilities. They expressed that they must pay between 1,000 (1 USD) and 5,000 naira (5 USD) before receiving services, including fast-track in some instances. These high costs may act as barriers to accessing care in their preferred models and negatively influence their willingness and experiences with these services. As one of the respondents put it
*"The fees at facility XX have been a problem, especially since I travel from my town with my daughter to collect my medication there. Because of the charges, I requested a transfer to another facility for treatment."*

**Attitude of healthcare workers and confidentiality**
The most significant negative determinants for the young persons and key population were confidentiality and poor attitude of healthcare workers. These participants expressed that their negative experiences stemmed from negative attitudes of healthcare workers, including stigmatizing behavior characterized by unfriendly demeanor, lack of empathy, and disrespectful behavior by these healthcare workers. As one of the respondents put it

> "*Yes. They are harsh. Sometimes, they will be harsh to you, and I will be like, I was not the one that put myself in this situation, so you can't be harsh to me because of my condition because life goes around. You do not know where you will meet me, and I will help.*"

Another factor that negatively influenced participants' experience at the DSD service delivery points was the fear of accidental disclosure. This results when service providers knowingly or unknowingly disclose recipients of care status to others without their consent. An example was given by one of the participants who said

"*I know someone who works as a medical records staff at the Federal Medical Center. One day, I found myself in the same community where he lives. He called me, we greeted each other, and I left. After I left, he told someone, 'Do you see that guy? He collects drugs at FMC.' The other person came and mentioned it to me.* "

### Theme 3: Suggestions on How to Improve DSD Services

In exploring suggestions to enhance Differentiated Service Delivery (DSD) services for HIV care, participants provided valuable insights into various strategies to improve service quality and accessibility. The themes highlighted encompass a wide range of recommendations, each addressing specific areas of improvement within the healthcare system. Notably, respondents underscored the importance of enhancing healthcare worker training to ensure proficiency in delivering DSD services (Training of HCW), aligning healthcare practices with the specific needs of people living with HIV (Engaging HCW in tune with the needs of PLHIV), and increasing awareness about DSD among PLHIV and healthcare workers.

**Training of Healthcare Workers (HCW)**

Respondents underscored the critical need for comprehensive and ongoing training programs tailored to healthcare workers to ensure their proficiency in delivering Differentiated Service Delivery (DSD) services. They emphasized the importance of equipping HCWs with the necessary skills, knowledge, and competencies to effectively address the unique needs of people living with HIV (PLHIV) within the DSD framework.

**Engagement of Healthcare Workers (HCW) who are PLHIV**

Engaging healthcare workers (HCWs) who deeply understand the needs of the recipients of care is crucial for fostering empathy, understanding, and peer support in facility and community service delivery points. This is especially important for the key population and young persons. HCWs with lived experience of HIV bring a unique perspective that enhances patient-provider relationships, reduces stigma, and promotes inclusivity. Their visibility as role models and advocates helps challenge misconceptions and discrimination, while peer support initiatives contribute to improved treatment outcomes and patient empowerment. Overall, the engagement of HCWs who are PLHIV plays a vital role in advancing patient-centered HIV care and promoting a supportive healthcare environment.

**DSD Awareness Programs**

Individuals stressed the significance of implementing robust and targeted awareness programs to enhance PLHIVs' understanding and uptake of DSD services. They emphasized the need for comprehensive educational campaigns aimed at PLHIVs and healthcare providers to promote awareness, dispel myths, and foster informed decision-making regarding DSD participation.

**Discussion**

In this study, we identified barriers and enablers to accessing DSD services by PLHIV and matched this with healthcare workers' perceptions regarding DSD and clients' preferences. The study showed that both healthcare workers and PLHIV viewed DSD as an acceptable treatment option that can lead to positive outcomes and that convenience, cost considerations, and healthcare worker attitudes are key factors that influence PLHIV experiences with DSD and can affect the decision to be devolved and retained in DSD.

Convenience of the PLHIV emerged as a key consideration for healthcare providers and clients in choosing DSD models. This correlates with findings from studies in Nigeria,[15] and Ghana.[16] The convenience of having drugs delivered to their homes or picked up at their preferred time from a pharmacy was a significant consideration that influenced a positive experience for the PLHIV in these studies. While the Nigerian and Ghana studies focused on a narrower demographic of PLHIV, our study explored the perception of a wider PLHIV group. While the study from Ghana focused on care recipients from a single facility, the Nigerian study focused on persons devolved to DSD in Northern Nigeria. We explored the experience of persons devolved across other regions of Nigeria including the North. This enables us to explore the social and demographic characteristics that can affect devolvement on a larger scale.

Cost had both a positive and negative effect on clients experience on DSD. While reducing the transport cost positively influenced the DSD experience, payment at the DSD service points resulted in a significant negative experience for PLHIV. Women were more vocal about cost considerations, which could be linked to the reduced financial independence experienced by women in this setting.[17] A study in Uganda among Female sex workers assessing DSD also identified the cost of transport to health facilities as a barrier to the uptake of HIV services from the healthcare facilities and positively influenced their acceptance of DSD.[18] While this study focused exclusively on One of the priority populations for HIV; Female sex workers, our study explores the attitudes of Female PLHIV in the general population. These populations' needs and experiences vary, which can influence their DSD experience.

This study also identified concerns around confidentiality and the risk of stigma as additional factors influencing participant experience with DSD. The risk of accidental disclosure was a barrier to accessing care in community-based DSD models. This risk was heightened by poor healthcare worker attitudes during community drug delivery. The Studies in Nigeria[15] and Uganda[18] referenced above also identified stigma as a barrier to accessing services in the community. Similar to the findings in the Ghana study, inadvertent disclosure during out-of-facility service delivery was one of the major sources of stigma to the PLHIV. Educational activities targeted at healthcare workers, recipients of care, and the general population may be of help in addressing these issues regarding stigma.[19,20]

The study identified the need to continuously build healthcare worker capacity on client engagement as one recommendation for improving services at the DSD service delivery points. This training will improve healthcare workers' ability to deliver quality services and build their capacity in non-discriminatory service provision. This may involve training healthcare workers on cognitive behavioral therapy and providing mental health and psychosocial support services.

At the program planning level, additional efforts should be made to advocate for removing or subsidizing service charges at DSD service delivery points. This will increase the acceptance of these services and reduce the financial burden on persons accessing DSD services. While a case could be made for leaving the service charge at these DSD sites, further analysis needs to be done to determine the optimal amount to be paid at these sites and the modalities for payment. If it is determined that these payments are essential, a payment option could be to incorporate these costs as part of routine healthcare costs covered by national health insurance; this will improve accessibility and further streamline the payment processes.

**Conclusion**

The findings of this study highlight the multifaceted factors influencing individuals' experiences and perceptions of DSD models. While there is considerable awareness and acceptance of DSD services among participants,

several challenges and areas for improvement exist. These challenges as perceived PLHIV include but not stigma, cost of care, and attitude of Healthcare workers.

Addressing these challenges will require a concerted effort from healthcare providers, policymakers, and PLHIV. Strategies to enhance healthcare worker training, optimize service delivery points to promote confidentiality, reduce financial barriers, and streamline service delivery PLHIV are crucial for optimizing DSD models and ensuring equitable access to HIV care and treatment. Furthermore, prioritizing patient-centered approaches and engaging with community-based organizations and peer networks will be essential for reaching marginalized populations and addressing gaps in HIV education and awareness. By addressing these challenges and implementing targeted interventions addressing confidentiality, accessibility, and affordability, Nigeria can strengthen its HIV service delivery system and improve health outcomes for individuals living with HIV across Nigeria.

## REFERENCES


1. UNAIDS. Global AIDS Update. Geneva: UNAIDS; 2020.
2. National Agency for the Control of AIDS (NACA): National Guidelines for HIV Prevention, Treatment, and Care 2016. Available at https://www.naca.gov.ng/wp-content/uploads/2017/08/National-HIVAIDS-Prevention-and-Treatment-Guidelines_2016.pdf
3. Jwanle P, Ibiloye O, Obaje M, Ngwoke K, Usha T, Amoo O, Ogunsola O, Okezie U, Olaitan R, Ofuche E, Onwuatuelo I, Samuels J, Fagbamigbe J, Nwagagbo F, Ogbanufe O, Okoye M, Okonkwo P. Accelerating HIV epidemic control in Benue state, Nigeria, 2019-2021: the APIN program experience. Ther Adv Infect Dis. 2023 Feb 18;10:20499361231153549. Doi: 10.1177/20499361231153549. PMID: 36814516; PMCID: PMC9940220.
4. Boyd AT, Ogbanufe O, Onyenuobi C, Mgbakor I, Bachanas P, Olupitan O, Umeh C, Adegboye A, Owhonda G, Odafe S, Jahun I, Dakum P, Mensah C, Gwamna J, Onotu D, Dirlikov E, Williams-Sherlock M, Okolo C, Verinumbe T, Idakwo S, Kumtap MU, Ellerbrock T, Swaminathan M. Scale-up of antiretroviral treatment access among people living with HIV in Rivers State, Nigeria, 2019--2020. AIDS. 2021 Jun 1;35(7):1127-1134. doi: 10.1097/QAD.0000000000002858. PMID: 33946087; PMCID: PMC8366506.
5. CDC: 2021 "HIV treatment surge in Nigeria doubles the number of people on treatment in two years." https://www.cdc.gov/globalhivtb/who-we-are/success-stories/success-story-pages/treatmentsurgenigeria.html
6. UNAIDS (2021a) Differentiated HIV testing and treatment service in Africa, UNAIDS. Available at: https://www.unaids.org/en/resources/presscentre/featurestories/2021/december/20211210_differentiated-service-delivery (Accessed: 09 May 2023)
7. Roy M, Bolton-Moore C, Sikazwe I, Holmes C. A review of differentiated service delivery for HIV treatment: Effectiveness, mechanisms, targeting, and scale. Curr HIV/AIDS Rep. 2019;16(4):324-334. Available from: https://doi.org/10.1007/s11904-019-00454-5
8. UNAIDS. Differentiated service delivery for HIV. Geneva: UNAIDS; 2016. https://www.differentiatedservicedelivery.org/wp-content/uploads/9789240023581-eng-1.pdf
9. Attah, M. Implementing multi-month scripting (MMS) for ART refills in Nigeria Consultation on differentiated ART delivery in WCA Background on Nigeria. Retrieved March www.iasociety.orgwww.iasociety.org (Accessed: 09 May 2023).
10. Boyd A, Jahun I, Dirlikov E, Greby S, Odafe S, Abdulkadir A, et al. Expanding access to HIV services during the COVID-19 pandemic—Nigeria, 2020. AIDS Res Ther. 2021;18(1). Available from: https://doi.org/10.1186/s12981-021-00385-5
11. The WHO "Nigeria's early adoption of Differentiated Services Delivery model buffers effect of COVID-19 pandemic among People Living with HIV"https://www.afro.who.int/news/nigerias-early-adoption-differentiated-services-delivery-model-buffers-effect-covid-19
12. Boyd A, Ogbanufe O, Onyenuobi C, Mgbakor I, Bachanas P, Olupitan O, et al. Scale-up of antiretroviral treatment access among people living with HIV in Rivers State, Nigeria, 2019--2020. AIDS. 2021;35(7):1127-1134. Available from: https://doi.org/10.1097/qad.0000000000002858

13. Ehrenkranz P, Grimsrud A, Holmes CB, Preko P, Rabkin M. Expanding the Vision for Differentiated Service Delivery: A Call for More Inclusive and Truly Patient-Centered Care for People Living With HIV. Journal of Acquired Immune Deficiency Syndromes. 2021 Feb 1;86(2):147-152. PMID: 33136818; PMCID: PMC7803437.
14. Stafford KA, Odafe SF, Julia Lo, Ibrahim R, Ehoche A, Niyang M, Aliyu G, Gobir B, Onotu D, Oladipo A, Dalhatu I, Boyd AT, Ogorry O, Ismail L, Charurat M, Swaminathan M. Evaluation of the clinical outcomes of the Test and Treat strategy to implement Treat All in Nigeria: Results from the Nigeria Multi-Center ART Study. PLOS One, 2019, https://doi.org/10.1371/journal.pone.0218555
15. Semo B-w, Ezeokafor N, Adeyemi S, Kpamor Z, Mugo C (2023) Differentiated service delivery models for antiretroviral treatment refills in Northern Nigeria: Experiences of people living with HIV and health care providers–A qualitative study. PLoS ONE 18(7): e0287862. https://doi.org/10.1371/journal.pone.0287862
16. Adjetey, V., Obiri-Yeboah, D. & Dornoo, B. Differentiated service delivery: a qualitative study of people living with HIV and accessing care in a tertiary facility in Ghana. BMC Health Serv Res 19, 95 (2019). https://doi.org/10.1186/s12913-019-3878-7
17. Central Bank of Nigeria: 2018. "Assessment of Women's Financial Inclusion in Nigeria" available at https://www.cbn.gov.ng/Out/2020/DFD/Assessment%20of%20Womens%20Financial%20Inclusion%20-%20Exec%20Summary.pdf assessed 6th May 2024.
18. Atuhaire, L., Shumba, C.S. & Nyasulu, P.S. "My condition is my secret": perspectives of HIV positive female sex workers on differentiated service delivery models in Kampala Uganda. BMC Health Serv Res 22, 146 (2022). https://doi.org/10.1186/s12913-022-07561-x
19. Machowska A, Bamboria BL, Bercan C, Sharma M. Impact of 'HIV-related stigma-reduction workshops' on knowledge and attitude of healthcare providers and students in Central India: a pre-test and post-test intervention study. BMJ Open. 2020 Apr 12;10(4):e033612. doi: 10.1136/bmjopen-2019-033612. PMID: 32284388; PMCID: PMC7201299.
20. Sommerland N, Masquillier C, Rau A, Engelbrecht M, Kigozi G, Pliakas T, Janse van Rensburg A, Wouters E. Reducing HIV- and TB-Stigma among healthcare co-workers in South Africa: Results of a cluster randomized trial. Soc Sci Med. 2020 Dec;266:113450. doi: 10.1016/j.socscimed.2020.113450. Epub 2020 Oct 17. PMID: 33126096.

Data

Click here to access/download
**Supporting Information**
Copy of KII Data.xlsx

Data

Click here to access/download
**Supporting Information**
Copy of DPR_Qualitative_FGD_-_all_versions_-_labels (1).xlsx

Data

Click here to access/download

**Supporting Information - Compressed/ZIP File Archive**

FGD Transcripts.zip
