# Supplementary material for: Perception of People Living with HIV and Healthcare Workers on Differentiated Service Delivery Programs in Nigeria: A Qualitative Study
Source: PLoS One. 2025 May 27;20(5):e0309254. doi: 10.1371/journal.pone.0309254 (PMC12111639; doi:10.1371/journal.pone.0309254)
Supplement: Attachment — Submitted filename: Rebuttal Letter_DSD Perspective Study.docx24Nov24.docx [file pone.0309254.s003.docx]

Adewale Akinjeji

Health Systems and Policy (HSP) research group

Department of Global Public Health, Karolinska Institutet

22/11/2024

Mabel Kamweli Aworh, DVM, MPH, PhD, FCVSN

Academic Editor

PLOS ONE

Dear Dr. Aworh,

Re: Manuscript PONE-D-24-32833

Thank you for the opportunity to revise and resubmit our manuscript, titled "Perception of People Living with HIV and Healthcare Workers on Differentiated Service Delivery Programs in Nigeria: A Qualitative Study." We appreciate the constructive feedback from the reviewers and editorial team, which has helped us refine the manuscript significantly. Below, we provide a point-by-point response to the comments and outline the corresponding revisions made in the manuscript.

Reviewer #1

Methodology:

Comment: Justification for selecting the four states was not provided.

Response: We have included an explanation of the selection criteria, highlighting the socio-cultural diversity and HIV prevalence rates that influenced the choice of states.

Comment: Clarify key populations and stratification details.

Response: The manuscript now defines key populations explicitly and provides justification for the stratification approach used.

Comment: Details about the content of the semi-structured interview guide were vague.

Response: We have expanded on the guide's content and included key themes covered during the interviews.

Results:

Comment: Include numerical representation of qualitative data.

Response: Numerical summaries for participant experiences have been incorporated where applicable, alongside exemplar quotes.

Reviewer #2

Limitations and Future Work:

Comment: Explicit discussion of study limitations and future research directions is needed.

Response: A dedicated section on limitations has been added, alongside suggestions for future research.

Ethical Considerations:

Comment: Ensure ethical guidelines regarding participant confidentiality and informed consent are addressed.

Response: We have elaborated on the ethical considerations, emphasizing the processes for obtaining informed consent and ensuring participant confidentiality.

Reviewer #3

Abstract:

Comment: Rephrase certain sentences for clarity.

Response: We have rephrased the specified sentences in the abstract for consistency and precision.

General Formatting:

Comment: Ensure consistent formatting of in-text citations and adherence to PLOS guidelines.

Response: All citations have been revised to align with PLOS guidelines, and continuous line numbering has been added.

Introduction and Discussion:

Comment: Provide references for statements and enhance discussions.

Response: References for all key statements have been added, and the discussion has been expanded to include more relevant literature.

Reviewer #4

Background Information:

Comment: Expand on DSD models and define key terms.

Response: We have provided additional context on DSD models and clearly defined terms such as “healthcare workers” and “key populations.”

Findings and Discussion:

Comment: Include demographic data breakdown and quotes for KII.

Response: Demographic data tables have been reorganized, and representative quotes from KIIs have been added.

Comment: justify the reason for excluding virally unsuppressed persons from the study

Response: We included only individuals who achieved viral suppression, as this is a key criterion for devolvement into differentiated service delivery (DSD) models in Nigeria. Including those ineligible for DSD services would contradict the study's aim of understanding the perspectives of care recipients on DSD and could introduce bias, potentially skewing the data

We believe these revisions address all comments and significantly strengthen the manuscript. A marked-up copy with track changes and a clean version are included with this submission.

We thank the reviewers and editorial team for their thoughtful insights, which have greatly improved our work. Please do not hesitate to reach out if further clarification or additional revisions are required.

Sincerely,

Akinjeji Adewale

On behalf of all co-authors
